# Understanding the utilisation of a novel interactive electronic medication safety dashboard in general practice: a mixed methods study

**DOI:** 10.1186/s12911-020-1084-5

**Published:** 2020-04-17

**Authors:** Mark Jeffries, Wouter T. Gude, Richard N. Keers, Denham L. Phipps, Richard Williams, Evangelos Kontopantelis, Benjamin Brown, Anthony J. Avery, Niels Peek, Darren M. Ashcroft

**Affiliations:** 1 Centre for Pharmacoepidemiology and Drug Safety, Division of Pharmacy and Optometry, School of Health Sciences, University of Manchester, Manchester, UK; 2 NIHR Greater Manchester Patient Safety Translational Research Centre, University of Manchester, Manchester Academic Health Sciences Centre (MAHSC), Manchester, UK; 3 Amsterdam UMC, University of Amsterdam, Department of Medical Informatics, Amsterdam Public Health Research Institute, Amsterdam, The Netherlands; 4 Health eResearch Centre, School of Health Sciences, University of Manchester, Manchester, UK; 5 NIHR School for Primary Care Research, University of Manchester, Manchester, UK; 6 Division of Primary Care, University of Nottingham, Nottingham, UK

**Keywords:** Information technology, Medication safety, Prescribing, Primary care, Clinical pharmacy

## Abstract

**Background:**

Improving medication safety is a major concern in primary care settings worldwide. The Salford Medication safety dASHboard (SMASH) intervention provided general practices in Salford (Greater Manchester, UK) with feedback on their safe prescribing and monitoring of medications through an online dashboard, and input from practice-based trained clinical pharmacists. In this study we explored how staff working in general practices used the SMASH dashboard to improve medication safety, through interactions with the dashboard to identify potential medication safety hazards and their workflow to resolve identified hazards.

**Methods:**

We used a mixed-methods study design involving quantitative data from dashboard user interaction logs from 43 general practices during the first year of receiving the SMASH intervention, and qualitative data from semi-structured interviews with 22 pharmacists and physicians from 18 practices in Salford.

**Results:**

Practices interacted with the dashboard a median of 12.0 (interquartile range, 5.0–15.2) times per month during the first quarter of use to identify and resolve potential medication safety hazards, typically starting with the most prevalent hazards or those they perceived to be most serious. Having observed a potential hazard, pharmacists and practice staff worked together to resolve that in a sequence of steps (1) verifying the dashboard information, (2) reviewing the patient’s clinical records, and (3) deciding potential changes to the patient’s medicines. Over time, dashboard use transitioned towards regular but less frequent (median of 5.5 [3.5–7.9] times per month) checks to identify and resolve new cases. The frequency of dashboard use was higher in practices with a larger number of at-risk patients. In 24 (56%) practices only pharmacists used the dashboard; in 12 (28%) use by other practice staff increased as pharmacist use declined after the initial intervention period; and in 7 (16%) there was mixed use by both pharmacists and practice staff over time.

**Conclusions:**

An online medication safety dashboard enabled pharmacists to identify patients at risk of potentially hazardous prescribing. They subsequently worked with GPs to resolve risks on a case-by-case basis, but there were marked variations in processes between some practices. Workload diminished over time as it shifted towards resolving new cases of hazardous prescribing.

## Background

The safe prescribing and monitoring of medicines is an important aspect of health care provision worldwide as recently emphasised by the latest WHO Global Patient Safety Challenge (2017) “Medication without harm” [1]. In a retrospective study of prescribing over a period of 12 months in 15 English general practices, the prevalence of monitoring or prescribing errors in over 6000 unique prescription items was found to be 4.9%, with 18.7% of patients receiving at least one prescribing or monitoring error [2]. In studies investigating the prevalence of hazardous prescribing in primary care in the UK using prescribing safety indicators, it was found that between 5.2 and 5.5% of patients received potentially hazardous prescriptions and 7.6–11.8% of patients had not received recommended monitoring tests, though there was marked variation between general practices [3, 4].

Information technology (IT) based tools have been utilised in supporting general practitioners and other health professionals to improve the quality and safety of prescribing [5, 6]. For medication safety this typically involves computerised decision support (CDS) at the point of care [7]. However, CDS tends to suffer from two problems: firstly, systems often do not fit into clinical workflow since they interrupt to correct decisions that have already been taken, and secondly some systems have been seen to generate too many alerts which causes ‘alert fatigue’ and a drop in effectiveness after initial positive results [8]. These problems eventually cause some clinicians to simply ignore all alerts [9, 10]. The pharmacist-led information technology intervention for medication errors (PINCER) intervention provided computer-generated feedback about patients potentially exposed to hazardous prescribing [11]. In contrast to CDS systems, this feedback took place after the clinical encounter and therefore did not suffer from those same problems [12]. The PINCER trial demonstrated that pharmacists working collaboratively with primary care physicians to act upon the feedback reduced the numbers of those affected by particular prescribing safety indicators compared with the provision of feedback without pharmacist visits [11]. Pharmacists were trained in educational outreach and root cause analysis techniques so that they could identify, resolve and prevent potentially hazardous prescribing and drug monitoring in partnership with local staff at general practices [13]. The intervention highlighted the pivotal role of pharmacists in the intervention’s effectiveness. There were, however, indications that the reductions in risk due to PINCER may be temporary because the intervention did not necessarily sustain reductions in incidents of hazardous prescribing for all indicators beyond 6 month follow up [11]. This was in part because the feedback comprised a single report of data extracted from local electronic health records, whereas multiple cycles of feedback would typically be more effective [14, 15]. The PINCER evaluation identified the importance of integration of the pharmacists into the practice team particularly in implementing changes however it did not investigate exactly how the computer-generated feedback was used by stakeholders [16].

The **S**alford Medication safety dASHboard (SMASH) intervention extends PINCER by providing feedback through an electronic, interactive medication safety dashboard alongside support from practice based pharmacists trained in root cause analysis [17]. The dashboard, that is refreshed daily, provides feedback about identified patients exposed to potentially hazardous prescribing and inadequate blood-test monitoring. A previous qualitative study specifically explored the ways in which the full SMASH intervention was adopted, implemented and embedded in general practices and revealed that the success of this work was critically dependent upon the pivotal role of the pharmacist and how they and other clinicians and staff members interacted with each other to create a learning health system [18]. This previous qualitative study focused on the ways in which the intervention was integrated into practice and drew upon Normalisation Process Theory to understand this process [19].

Interventions based on digital technology such as SMASH are complex interventions [20–22] that facilitate quality improvement by providing local teams with tools to measure and monitor their performance and take appropriate action. It will often depend on the organisational context, which aspects of clinical performance require improvement and how this is best achieved [23]. This complexity resides among other things in the interaction between various components of the intervention; people and technology can be reciprocally entwined within a sociotechnical system [24, 25]. Process evaluations are necessary to gain insight into why an intervention works or fails within different contexts and how it can be optimised [22, 26–31]. The electronic nature of the SMASH dashboard allows for new opportunities to study the mechanisms through which users interacted with the intervention quantitatively [32]. It has been recommended that qualitative and quantitative methods should be utilised together in process evaluations to gain a deeper understanding of complex pathways and identify the ways through which intervention components were operationalised and integrated within local settings [28–31].

Dashboards have been utilised in primary care and other health settings including for prescribing safety [33, 34]. A recent review concluded that while dashboards might improve clinical outcomes for patients the exact characteristics of dashboards and how they were utilised by clinicians was not fully understood [35]. In this study, we aimed to understand the specific ways in which clinical pharmacists and physicians interacted with the SMASH online dashboard and how the level of engagement with the dashboard varied between practices. By doing so this could enable us to understand how the SMASH intervention could be operationalised and integrated into practice and hence understand its impact and sustainability.

## Methods

### Study design

We adopted an integrated mixed-methods approach [36, 37]. We conducted semi-structured interviews with study participants, and we harnessed the user log files produced by the electronic dashboard in order to gain in depth insights into the ways the dashboard was utilised. The study was carried out alongside an interrupted time-series analysis evaluating the impact of the SMASH intervention in 43 general practices across Salford (a city in Greater Manchester, UK) which has a population of over 250,000. We used a mixed-methods study design, which involved synergistic utilisation of quantitative data from user interaction logs produced by the dashboard, and qualitative data from semi-structured interviews. Whilst the quantitative data was particularly used to assess the objective dashboard usage patterns across all practices, and the variation between those practices, the qualitative data was used to explain the patterns observed and explored how practices used the information extracted from the dashboard to resolve potential medication safety hazards; which would typically happen outside the dashboard.

### The SMASH intervention

Full details of the intervention have been published elsewhere [17]. Participating general practices within Salford Clinical Commissioning Group (CCG) received access to an electronic medication safety dashboard and support from clinical pharmacists working within the practices. The initial intervention period was 3 months but participants were free to continue using the dashboard after this time period. The dashboard utilised a set of prescribing safety indicators (Additional file 1) (e.g. the number of patients with a history of peptic ulcer or gastro-intestinal bleeding prescribed a non-steroidal anti-inflammatory drug (NSAID) without a gastro-protective medicine) and presents the resulting information to its users in both aggregated form and as lists of individual patients with potential safety hazards [11]. Five types of pages were available within the dashboard:

- Practice summary: practice size, number and percentage of patients currently affected by at least one indicator, and number of patients affected by multiple indicators. This is typically the landing page when accessing practice-specific data.
- Table overview: a table listing for each indicator the number and percentage of patients who are currently at risk, comparisons with the CCG average, and the trend over time. By default, the list of indicators was presented in descending order of the number of at-risk patients.
- Chart overview: same as the overview table but represented using bar and line charts.
- Patient lists: a list of NHS numbers of patients who are identified by the dashboard to be currently at risk for the selected indicator, including information about other indicators for which they are at risk and since when.
- Indicator information: explanation and evidence of the selected safety indicator and suggestions for taking corrective action; supported by references to scientific literature. In contrast to the other types of pages this was static information.

Clinical pharmacists were trained to work collaboratively with practice staff to resolve episodes of potentially hazardous prescribing and drug monitoring, and utilised root cause analysis techniques to identify and implement organisational ‘systems’ changes to prevent their future occurrence. Whereas some pharmacists were employed directly by a single practice and had already been in their role at the commencement of the intervention, others were employed by the local hospital trust and were aligned to groups of general practices in different neighbourhoods.

### Participants

Forty-three general practices participated in the SMASH intervention across the Salford Clinical Commissioning Group (CCG). All 44 general practices in Salford were eligible to participate in the study if they contributed to the Salford Integrated Record (SIR; a data warehouse containing primary care data from contributing practices across Salford). One practice wanted to participate in the SMASH intervention but was unable to do so as it did not contribute to SIR. Practices consented to take part in the study and began to receive the SMASH intervention thereafter [17]. Participants for the interviews were purposefully sampled from practices to reflect the different contexts in which SMASH was implemented, including variations in practice size, locality measures of social deprivation and type of electronic patient record software used (EMIS Web or VISION v3). General Practitioners (GPs), practice nurses, practice administrators and managers, and pharmacists working within each practice included were eligible for inclusion in the interviews. Individual participants were recruited on a purposive basis through direct contact with the pharmacist delivering the SMASH intervention at the practice(s) in question. Additional written informed consent was taken from individual participants prior to each interview, which included consent for the interviews to be audio recorded and transcribed verbatim and for anonymised quotations to be used in reports.

### Data collection and analysis

We extracted the quantitative data from user interaction logs produced by the dashboard during the first year of the intervention in each practice. The logs included page views, mouse clicks, hovers, and key strokes. Therefrom we determined the number and duration of interactions within practices, which pages (e.g. which indicators, which feedback modalities) were viewed, and under what circumstances that occurred. We compared the quantitative user interaction metrics between practices and user groups (e.g. pharmacists and practice staff) and over time using medians and interquartile ranges (IQRs) for non-normal distributions. We assessed the relationship between dashboard use and the number of patients potentially exposed to one or more medication safety hazards using Pearson’s correlation coefficient. Data analysis was undertaken using R statistical software version 3.3.0 (R Foundation for Statistical Computing).

The semi-structured interviews were carried out face-to-face by a qualitative health researcher (MJ) between April and December 2016. The researcher was not known to participants at the time of the interviews. Interviews were primarily conducted at the general practice where the participant was working. Five interviews were conducted at The University of Manchester and one at the Salford (CCG) offices. We analysed the qualitative interview data using a thematic template analysis approach [38]. A set of thematic codes was developed from independent reading of the first six interviews by three of the authors (MJ, RNK, DLP). This set of codes was applied to the all the transcripts using the NVivo version 10 qualitative data management software. In an iterative approach, the template was then further developed and adapted as the transcripts were coded. Finally, the coded extracts were analysed, and emerging themes highlighted which formed the basis of the results detailed below.

Quantitative and qualitative data were collected concurrently, analysed separately and then integrated in a process of using the quantitative and qualitative data to validate, explain, and complement their separate findings. This involved a strategy of synthesizing and weaving of the data based on the approach described by Fetters et al. (37), whereby the qualitative data was further explored to find explanations in the quantitative and vice versa.

## Results

The 43 Salford general practices started receiving the SMASH intervention between 18 April 2016 and September 2017. During the study period the dashboard logged 2626 sessions by 55 users (25 pharmacists and 30 practice staff). Twenty-five interviews were undertaken with 22 participants (12 pharmacists, four GPs, one practice nurse, one practice administrator, one practice manager and three CCG managers) working in 18 (42%) different general practices and at the CCG. One interview was conducted jointly (practice manager and practice administrator). Interviews lasted between 14 and 62 min, with three pharmacists and one GP interviewed longitudinally on two occasions in order to capture how the intervention might be sustained (see Additional file 1).

### Interactions with the dashboard

#### Primary users of the dashboard and frequency of use

Table 1 presents the frequency and duration of interactions with the dashboard in the participating practices throughout the first year of receiving the SMASH intervention. Practices interacted with the electronic dashboard with a median average frequency of 6.6 (IQR, 4.2–9.3) times per month. The dashboard was primarily used by pharmacists who interacted a median of 5.9 (IQR, 3.3–8.2) times per month as opposed to local practice staff who interacted 0.5 (IQR, 0–1.5) times per month.

**Table 1:** Frequency and duration of dashboard interactions in the 43 participating general practices in Salford during the first year of the SMASH intervention. All presented data are medians (interquartile range) across practices

	Median average number of views per month (IQR)	Median average time spent in minutes per month (IQR)	Median total number of at-risk patients (IQR)
Users			
Any user	6.6 (4.2–9.3)	113.8 (74.1–183.2)	
Pharmacists	5.9 (3.3–8.2)	104.7 (51.2–136.0)	
Practice staff	0.5 (0.0–1.5)	5.3 (0.0–27.6)	
Time			
First quarter	12.0 (5.0–15.2)	217.4 (108.4–319.6)	
Second quarter	4.5 (2.8–7.4)	69.6 (37.7–102.8)	
Third quarter	6.3 (3.7–10.2)	90.4 (50.0–172.2)	
Fourth quarter	5.7 (2.7–9.3)	88.4 (27.4–155.9)	
Dashboard pages			
Practice summary	9.1 (5.3–14.2)	181.5 (66.9–289.0)	
Table overview	18.4 (12.2–28.9)	765.9 (380.6–1132.9)	
Chart overview	0.9 (0.4–3.0)	25.2 (4.6–119.2)	
Indicator information	0.5 (0.2–1.0)	67.0 (4.5–228.8)	
Patient list (any medication safety indicator)	19.3 (14.1–35.6)	2907 (1722.0–5371.3)	67 (45–128.5)
Patient lists for specific types of medication safety risks			
1. Prescription of an oral NSAID without co-prescription of an ulcer-healing drug in a patient aged ≥65 years	4.4 (2.5–9.0)	747.9 (338.9–1307.8)	26 (11.5–42.5)
2. Prescription of an oral NSAID without co-prescription of an ulcer-healing drug to a patient with a history of peptic ulceration	0.3 (0.1–1.0)	13.1 (0.4–92.2)	1 (1–3)
3. Prescription of an antiplatelet drug without co-prescription of an ulcer-healing drug to a patient with a history of peptic ulceration	2.0 (0.5–2.8)	224.4 (98.1–456.4)	4 (2–6.5)
4. Prescription of warfarin or NOAC in combination with an oral NSAID	1.3 (0.5–1.9)	98.7 (39.3–211.7)	4 (2–6)
5. Prescription of warfarin or NOAC in combination with an antiplatelet drug without co-prescription of an ulcer-healing drug	1.3 (0.8–2.5)	130.3 (77.5–358.2)	4 (2–9)
6. Prescription of an aspirin in combination with another antiplatelet drug without co-prescription of an ulcer-healing drug	2.5 (1.1–3.8)	345.5 (142.6–532.6)	8 (5–13)
7. Prescription of a non-selective beta-blocker to a patient with asthma	3.8 (2.0–5.5)	600.3 (358.0–914.2)	15 (8–28.5)
8. Prescription of a long-acting beta-2 inhaler (excluding combination products with inhaled corticosteroid) to a patient with asthma who is not also prescribed an inhaled corticosteroid	0.8 (0.0–1.7)	65.2 (0.0–188.6)	2 (0–6)
9. Prescription of an oral NSAID to a patient with heart failure	0.8 (0.3–1.8)	50.9 (5.5–123.0)	2 (1–5)
10. Prescription of an oral NSAID to a patient with chronic renal failure (eGFR< 45)	0.5 (0.2–1.3)	55.7 (0.8–114.8)	3 (1.5–5)
11. Prescription of methotrexate without both a recent full blood count and a recent liver function test	1.2 (0.7–2.3)	158.2 (70.3–315.1)	4 (2.5–6)
12. Prescription of amiodarone without a thyroid function test	1.5 (0.8–3.3)	191.7 (40.3–358.3)	4 (2–6)
Multiple medication safety indicators	0.3 (0.1–0.7)	17.5 (0.8–158.0)	4 (2–7)

*NSAID* nonsteroidal anti-inflammatory drug. *NOAC* novel anticoagulants such as apixaban, dabigatran or rivaroxaban. *eGFR* estimated Glomerular Filtration Rate. Antiplatelet drugs are aspirin, clopidogrel, prasugrel and ticagrelor. Peptic ulceration includes upper gastrointestinal bleeds, but does not include peptic ulcer surgery, gastritis, duodenitis or oesophageal varices. Ulcer-healing drugs include the PPIs and H2-antagonist – it does not include misoprostol, sucralfate or bismuth

Both practice staff and pharmacists considered the dashboard as something for the pharmacist to use. Whilst pharmacists sought to encourage GPs to use the dashboard, the GPs themselves made decisions on whether they needed a user account based on whether they thought they would access the dashboard:

*“I have not seen anyone look at the dashboard, apart from the, I think the practice manager may have access to it, I’m not sure. But I’ve not seen them. The only way they find out about the dashboard is; through me” (Pharmacist –P7)*

*“…we’ve only got one lead GP [and] four associate GPs so they don’t…it depends on what they want to get involved with. So, they’re not salaried so they can’t just be given all of the work for the GP practice. It’s very much at their discretion what projects they want to get involved with and if they don’t want to get involved, they don’t want to get involved.” (Pharmacist -P1)*

Similarly, variation in use of the dashboard was also described by one GP who explained that whilst some GPs at their practice perceived they would not use the dashboard, others had access to view evidence summaries or made decisions to take the lead on the use of the dashboard.

*“So, at the meeting we said who wants access to this? We’ve said the people that want access I've given them the email link so that they can login. Some people (GPs) have just said, they don't want access, […] there is no point because I'm not going to look at it. Then a couple of people have said I want to be able to get these. I want to be able to get the evidence for the educational point of view*.” *(GP1)*

**Fig. 1:**
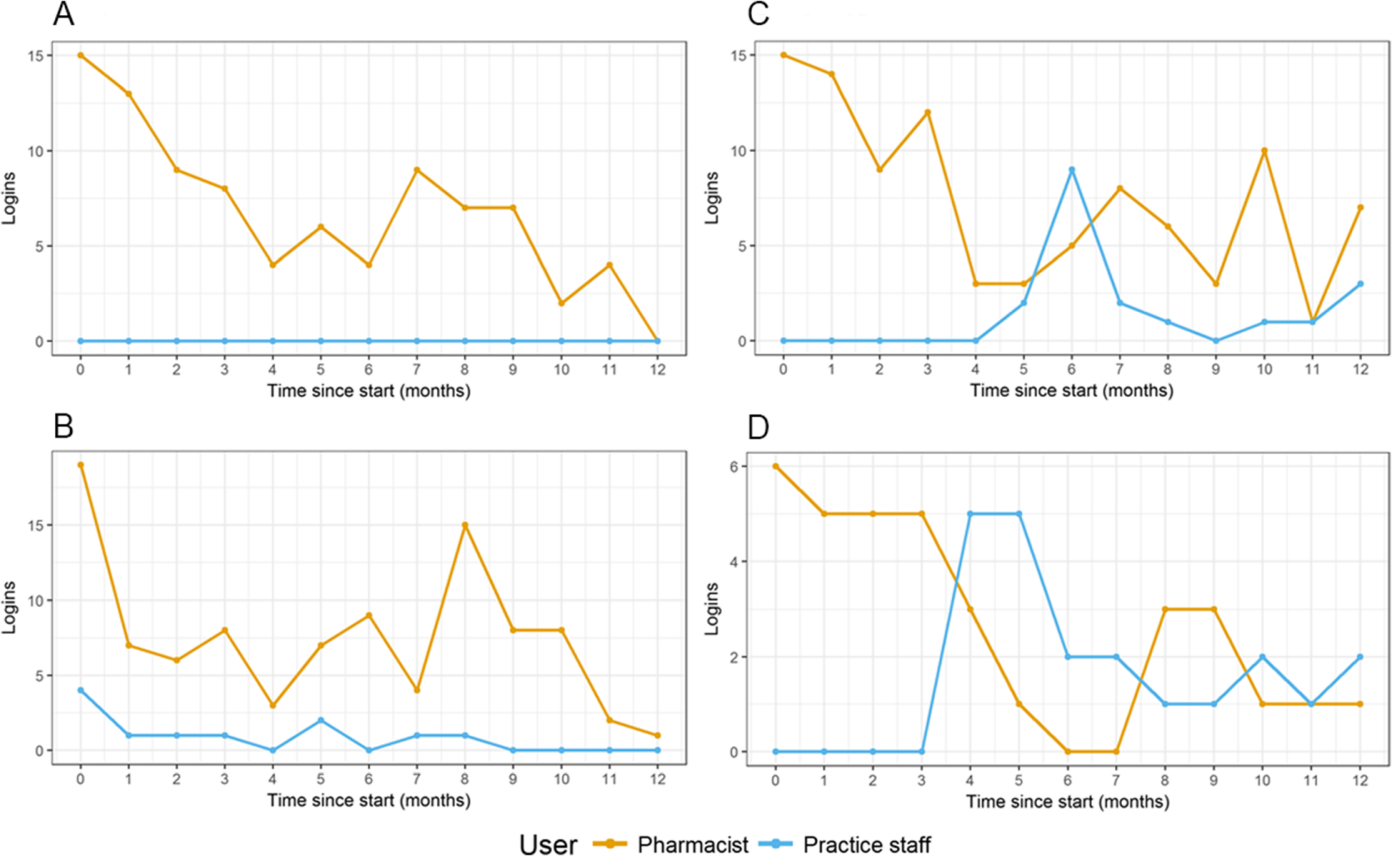
SMASH dashboard logins by pharmacists and local practice staff per month. Number of SMASH dashboard logins by pharmacists and local practice staff per month, over the first 12 months of the intervention, in four different general practices. The four practices were chosen to illustrate the variety of patterns found across all 43 participating general practices

Figure 1 presents examples of typical user patterns found in individual practices concerning the frequency of dashboard interactions by pharmacists and practice staff over time. Although all practices had created user accounts for practice staff to access the dashboard, in 24 (56%) practices non-pharmacy staff never or hardly interacted with it (e.g. Fig. 1a and b). Pharmacists experienced barriers to using the dashboard as a result of competing priorities in the practice relating to tasks not directly associated with the intervention, such as repeat prescription requests or medicine reconciliations of patients discharged from the hospital. One such practice pharmacist described her role as involving a range of such activities;

*“I end up having my fingers in a few different pies. I look after clinical post and letters, transfer of care out of hospital, so I do medicines reconciliation kind of back out to the GP side of things. I do telephone calls, so I’ve got my own telephone clinic where patients with medication queries, problems, supply issues rather than going through to the GP they now come through to myself to sort out. [...] So I do a lot of medicines information queries for the doctors about doses availability of treatment, NICE guidance and the same for patients if they ring up as well.” (Pharmacist -P1)*

As a consequence, they *“would need to take some of my time out of the day to particularly look at the SMASH dashboard.” (Pharmacist -P1)*

The frequency with which practice staff, including pharmacists, interacted with the dashboard declined over time (Fig. 1). While during the first 3 months the median average frequency of dashboard interactions was 12 (IQR, 5–15.2) times per month; during the rest of the study period this was 5.5 (IQR, 3.5–7.9) times per month (Table 1). This pattern was consistent with the way pharmacists described using the dashboard in that they would initially investigate all the patients already highlighted as being potentially at risk from hazardous prescribing, and then once having reviewed those patients use the dashboard to investigate new emerging cases:

*“I think to start with it was very much an active tool to be used. I think its use will change, because I have certainly seen its use change from when we first got, like I say, when it was looking at the...we didn’t have massive numbers but, you know, the practice was looking at the fifty patients that were already affected by the indicator. Whereas, what we are doing now, looking at the new patients that are just coming on, it’s a different way of using the same tool.” (Pharmacist -P2)*

Similarly, other pharmacists reported checking the dashboard on *“probably, every week, two weeks, four weeks*,” *(Pharmacist-P12)* or to *“see if any new cases have come up and I check that on a monthly basis.” (Pharmacist -P7)*. Once the number of at-risk patients from one indicator was reduced, the pharmacist identified new cases as a way active surveillance of the prescribing in practice.

*“Well what I would like is for the pharmacist to keep using the dashboard to identify new cases, and then probably periodically for me or for the pharmacist to look at the graphs and just check that the graphs are not crawling back up again.” (GP1)*

Whereas in nearly all practices the overall dashboard interactions declined as pharmacists started using the dashboard less frequently, in 12 (28%) practices the interactions by the local staff excluding pharmacists increased (e.g. Fig, 1c and d). In 7 (16%) practices there was mixed use of the dashboard by both pharmacists and practice staff. This was reflected in the qualitative interviews in the perceptions of this practice pharmacist.

*“I think I can but I think it’s always going to remain with the pharmacist here. I don’t think there’s going to be a GP who suddenly becomes very interested in it and who decides to take SMASH off my hands and lead on SMASH here. It might happen at a bigger practice where you’ve got maybe a few more partners. You might be able to get a pharmacist and GP working alongside each other, so maybe together, once a month, they access a SMASH dashboard and sit together.” (Pharmacist -P1)*

**Fig. 2:**
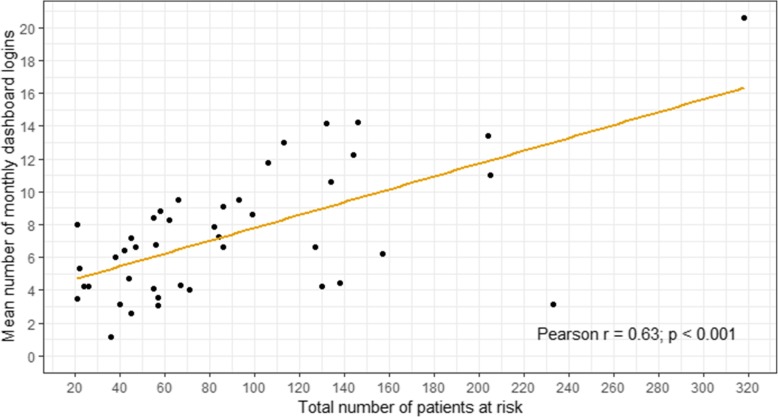
SMASH dashboard logins compared to the number of patients potentially exposed to medication safety hazards. Average number of SMASH dashboard logins across the 43 participating practices compared to the number of patients potentially exposed to one or more medication safety hazards during the first 12 months of the intervention

The frequency with which practices used the dashboard was correlated with the number of patients identified by the dashboard to be potentially exposed to one or more prescribing safety indicator hazards (Fig. 2; Pearson’s *r* = 0.63, *p* < 0.001).

#### Feedback modalities accessed, and areas of medication safety focused on by dashboard users

Users spent a median of 113.8 (IQR, 74.1–183.2) minutes per month interacting with the dashboard (Table 1); and had similar between-practice variation as the frequency of dashboard use. Dashboard sessions had a median duration of 14.3 (IQR, 3.9–29.7) minutes. During their interactions users predominantly viewed the practice summaries (9.1 [IQR, 5.3–14.2] times per month), table overview of all prescribing safety indicators (18.4 [IQR, 12.2–28.9] times per month), and lists of identified patients exposed to one or more indicators (19.3 [IQR, 14.1–35.6] times per month). Users scarcely accessed chart overviews and background evidence about the prescribing safety indicators.

Pharmacists typically started from the practice summaries and table overviews to gain an overview of the most urgent or common types of medication safety hazards and patients who were exposed to multiple indicator types identified by the dashboard, but also the ones that were ‘quick wins’ - hazards that were perceived as easier to resolve. This typically involved going back and forth between the table overview and various patient lists (i.e. for different medication safety indicators).

*“Yeah, so when I first started, they wanted me to focus on the people who were affected by multiple indicators first, because they deem those to be the most risky […] So then once we’d cracked those, then we went on…I did some of the easier ones, so the monitoring ones that are quite simple to sort out and then I’m in the middle of attacking the biggest one, so the 76 people are affected by the over 65s on NSAIDs…” (Pharmacist -P3)*

This corresponded with the finding that users spent most of their time viewing the patient lists of those prescribing safety indicators that had the highest number of at-risk patients (Table 1). This particularly concerned the indicators: ‘Prescription of an oral NSAID without co-prescription of an ulcer-healing drug in a patient aged ≥65 years’ (747.9 [IQR, 338.9–1307.8] minutes per month); ‘Prescription of a non-selective beta-blocker to a patient with asthma.’ (600.3 [IQR, 358–914.2] minutes per month); and ‘Prescription of aspirin in combination with another antiplatelet drug without co-prescription of an ulcer-healing drug’ (345.5 [IQR, 142.6–532.6] minutes per month). Over time the patient lists were viewed with lower frequency and duration as users focused upon looking for new cases.

### Workflow to resolve medication safety hazards identified by the dashboard

Once pharmacists and/or practice staff had identified patients from the various lists presented in the dashboard, a series of different activities related to medicines safety were initiated to address instances of high-risk prescribing. These different activities were characterised in three ways: by verifying information, in reviewing patients and decision making.

#### Verifying the information in the dashboard

Verification involved checking that the information in the dashboard was correct and checking the relevance or appropriateness of the alert. One pharmacist reported that they would *“check that actually what the dashboard is telling me is current” (Pharmacist -P10)* with regard to the patient’s repeat medications by cross referencing the dashboard with the patients’ health records. Similarly, another pharmacist spoke of making *“sure that each indication is valid” (Pharmacist -P2)*. Pharmacists verified the information in the dashboard against the appropriateness of the alert to the specific needs of patients and to *“make sure it was relevant” (Pharmacist P12).* This could involve decisions as to the appropriateness of changes to medications that patients had been on for many years and was seen as *“a bit awkward, trying to have that conversation for something that’s been longstanding for such a period of time”* (*Pharmacist* -*P3*). Pharmacists reflected upon the difficulties of assessing the appropriateness of prescribing or de-prescribing medicines. One pharmacist discussed how the practice they were working in had concerns about taking patients off medicines:

“…*they (the doctors) were a little bit concerned about us like taking everyone off NSAIDs without thinking about what alternative they were going to have for pain relief; because they are really effective drugs for like certain types of pain, so they were concerned that I was going to say, take all these people off NSAIDs and leave them in pain.” (Pharmacist -P5)*

Alternatively, another pharmacist reflected upon the appropriateness of adding to the patient’s medication burden.

*“…a patient on a PPI (Proton-pump inhibitor) they are on an NSAID, it just puts you in a direction of making an intervention that maybe isn’t necessary. So, it’s something that I have brought up, something that we need to look at from a training perspective for more junior pharmacists, that they are not just going in and starting PPI’s on patients without actually checking that the patient needs the NSAID. Because, potentially, what you were doing there is just adding to the polypharmacy.” (Pharmacist -P10)*

It was perceived that with such decisions to be made, it was important that “it (the use of the dashboard) needs to be clinically minded, well, clinical pharmacists who are using it” (pharmacist -P10)

#### Reviewing patient clinical records

Reviews involved working through those patients listed by the dashboard as being potentially at risk from hazardous prescribing and reviewing the patient’s electronic health records. Pharmacists described working through a list of those affected by a particular prescribing safety indicator in order to reduce the number of affected patients:

*"So […] I spend my time working my way throsgh each indicator, from that I look at each patient, find out the ins and outs of it, work out an action plan and then I will send that to the relevant person or take the appropriate action. So I might do things like ringing a patient up, getting doctors to write prescriptions, sending a letter for somebody to come in, documenting everything that I’ve found in the notes and then keep moving on to the next indicator" (Pharmacist-P3)*

This iterative process of going through the patient lists and reviewing the local records may explain that in practices throughout the study period users spent a median average of 2907 (IQR, 1722–5317) minutes, or 48.5 h, per month having opened a dashboard page containing patient lists.

The information in the dashboard became a starting point for conducting *“an initial look at the patient’s notes (to) then decide if there are issues on there, we need to contact the patient” (Pharmacist 6).* Review strategies required exploring the patient’s clinical record as described by this pharmacist;

*“So I’ve pulled off the data, put it onto a spreadsheet, gone through individually, gone through each individual NHS number[…], pull up the patient straight away and I’ve then gone into medicines, looked up why they are flagging, if I can’t see a reason why, then gone into the consultations and gone back through the history.” (CCG Pharmacist 3)*

The process of review, resulting from information in the dashboard, was further developed by pharmacists to undertake a wider and more holistic review of patients. Pharmacists described these actions as *“like some thought process of the history of the patient and what’s actually going on and why are they on these drugs.” (Pharmacist -P5)*. These more comprehensive reviews were seen as utilising the dashboard as a starting point that would allow further potential medicine issues to be explored.

*“So, we are using it as a way to guide us, in terms of what medication patients will focus on, but when we look at those patients, we’re obviously looking at the indicator that flags, but also making sure we look at the wider patient as well, because we don’t want to go in and just fix something, like order a blood test, and realise there’s other issues,[...] that also need resolving. So, we’re using it as a way of catching the patients, but then looking at the whole patient not just one particular indicator.” (Pharmacist -P6)*

#### Decision making

The use of the dashboard involved processes of communication and collaboration. Having explored the information contained in the dashboard, the pharmacists might pass this to GPs to make additional clinical decisions. This might involve brief verbal communication, email or electronically through the practice clinical patient record system (i.e. instant messaging). GPs were passed information to recommend changes to patients’ medicines or to review patients. One pharmacist felt it important to follow up a patient by having a discussion with the doctor.

*“And I went back to that practice this morning to speak to the GP about them because I didn’t want to just leave – it was nearly two sides of A4 of maybe we could do this – and not follow that up personally. [...] But I need to speak to the GP first because it’s quite an in-depth patient.” (Pharmacist - P9)*

The pharmacist and clinician might also work together to decide upon the *“best course of action”* (*Pharmacist -P5*). Such action planning required meeting with clinicians and built upon the findings within the dashboard

*“A few of the practices, I am covering four practices currently, so in two of those I am meeting with the GPs weekly to go through the findings from the dashboard, what I think the action should be and then we have agreed an action plan and then come up with a plan really.” (Pharmacist -P10)*

The engagement with GPs was interlinked with ways of accessing and interacting with the dashboard as this pharmacist reflected

*“Because by the time I had reviewed them I would have then had to make a decision, do I take those few patients to the GP now, or do I review all fifty patients and it take me two or three weeks to do it and then take them all. And the risk is that you forget the exact details of the original patients when the GP starts then saying, oh, as he got this condition, or what was his bloods doing or when...” (Pharmacist -P2)*

## Discussion

This study explored the interaction and engagement of healthcare staff with a medication safety dashboard in general practice. By understanding the frequency and duration of interactions, the variation of interactions between users and the workflows in the dashboard interactions we could explore how such tools may be utilised and the ways in which they might facilitate the long term sustainability of interventions to improve medication safety. We found that interaction with the SMASH dashboard varied markedly between general practices, with respect to the frequency of use, engagement by the range of practice staff and the extent to which engagement by pharmacists continued, or tailed off, over time. Practices with a higher number of at-risk patients used the dashboard more frequently. In half of the practices pharmacists were the sole users of the dashboard, whereas in the other half local practice staff also played a moderate to active role. Overall, the dashboard use transitioned over time from intensive use involving review of existing potential prescribing or monitoring hazards (starting with the most urgent and common ones), towards regular but less frequent checks of the dashboard to see if “new cases” required further investigation. The dashboard was utilised to improve medication safety by initiating three steps; once potentially at-risk patients were identified from the dashboard, pharmacists would typically respond by firstly verifying the risk by examining the patients’ electronic health records. They would then review the records in order to decide whether a change to the patient’s medication regiment was required before undertaking a decision-making process (often in collaboration with general practitioners) to resolve the risk.

Adaptation of workflow and work practices in response to information technology interventions in primary care has been highlighted elsewhere in the literature [8, 39–41]. How the SMASH dashboard was integrated into routine practice was an important finding of our study and was related to the variation in users and the differences in engagement by users. Pharmacists clearly engaged with the dashboard more than did GPs and other practice staff. This was related to the ways in which practices were able to adapt and evolve the intervention so it fitted well into their clinical workflow. The adaptation here typically entailed, particularly at the start of the intervention, the pharmacists working alone through lists of patients identified by the dashboard. Pharmacists in turn involved GP staff to make decisions with regard to the resolution of the hazard. As the high number of hazards and associated workload decreased, GP staff in some practices started using the dashboard primarily to check new cases. Pharmacists and GPs worked together to use the information in the dashboard to check records and make decisions on patients’ medications. Previous research has suggested that interventions utilising information technology can facilitate new working practices and collaborations between health professionals [42, 43]. Interactions and relationships affected the use of the dashboard particularly where communication in the form of feedback from the pharmacist accessing the system to the GPs ensured that medication safety actions were taken. This finding aligns with what Klecun has described as a *“sociotechnical network”* in which *“implementation is an ongoing social process influenced by stakeholders’ needs, interests, norms and ways of doing things”* [43]. Both this network and the dashboard allowed the pharmacists to utilise it for medication safety activities. A similar intervention to improve prescribing safety in primary care, which provided electronic feedback but did not involve pharmacists, found variations in implementation and differences in practice engagement, and that collective engagement within practices, particularly from clinicians, led to more successful implementation [44]. This variation might also be explained in that unlike the previous PINCER trial [11, 16] pharmacists in the SMASH intervention stayed with the practices beyond a three-month intervention period. As such this may be more reflective of how this intervention may be used and sustained in everyday practice in the longer term.

### Strengths and potential limitations

A particular strength and novelty of our study is the triangulation of qualitative and quantitative data. The quantitative data examined patterns of use of the dashboard across all participating practices, whereas the more in-depth qualitative data from a sub-sample off those practices helped to explain some of the findings observed from the quantitative data allowing for convergence of information from different sources. For example, the declining trend in the frequency with which the dashboard was used could be explained by pharmacists discussing the influence of competing priorities and how, after resolving more cases at the start of the intervention, they then would only check for new cases of hazardous prescribing. By adopting such a mixed-methods approach including interviews with a range of users at different time points in the intervention, we were able to understand how the intervention was used in practice.

A limitation is that fewer practice staff were interviewed than pharmacists. There were difficulties in recruiting GPs generally because of time pressures and workload, and because fewer numbers accessed the dashboard at all or as regularly as pharmacists. A limitation of analysing user log files is that we were unable to verify whether users logged into the dashboard with their own user accounts or whether multiple users (e.g. pharmacists together with a GP) were using the dashboard together on the same computer. Furthermore, in busy clinical practice users may have been distracted while logged into the dashboard to attend to other clinical work. This may have led to overestimating the amount of time users spent in the dashboard. However, we expect that the overestimation has been limited because users were automatically logged out 12 min after the last mouse click, and the session duration was calculated based on the last user action (e.g. a mouse hover) in the dashboard.

### Implications for further research, policy and practice

The important implications for this study are the ways in which use of medication safety dashboards such as SMASH can be integrated into practice. This integration and sustainability was evident in that the workload diminished over time, that users focused upon key areas in the dashboard at the start of the intervention period and in the ways the dashboard use integrated with workflows and work practices.

Using feedback to improve primary care medication safety can have many advantages over alerts by CDS systems, but requires pharmacist support which is resource-intensive, and may not have lasting effects across all outcomes, as demonstrated by the PINCER trial [11, 16]. The SMASH dashboard improves on PINCER by providing continuous rather than one-time feedback. The SMASH intervention required a high level of pharmacist input at the start of the intervention period followed by ongoing availability. Recent policy interventions to increase the availability of pharmacists working in general practices across England has helped to enhance the PINCER approach used in the SMASH intervention. The daily updates on patients exposed to potentially hazardous prescribing in the SMASH dashboard has helped to support medicines optimisation in line with the increased availability of pharmacists working in general practices. In addition, GP staff were also given access to the dashboard to enable them to engage with the intervention particularly as pharmacist involvement diminished following the more intensive initial intervention period; anticipating that the workload associated with improving medication safety would be much less by that time. Our study findings indicate that this workload reduced considerably over time, and once the initial high numbers of at-risk patient cases were resolved, users moved to less frequent surveillance for new cases. In some practices GP staff became involved in using the dashboard after pharmacist activity reduced. There was however considerable variation in practice staff involvement between practices; and in many, pharmacists still performed infrequent but regular checks of new cases of potentially hazardous prescribing identified by the dashboard. This implies that it would be feasible to make SMASH, and similar interventions in the future, sustainable in that they can continue to have effect after a resource-intensive period of pharmacist outreach since the dashboard routinely provides daily checks and the flexibility to work on key areas with the greatest need or highest risk.

The real-time information in the dashboard allowed users to recognise the new cases that required such resolution. That these were easily identifiable in the dashboard and that they were easily verified by users facilitated their resolution therefore helped to reduce user interactions to manageable and sustainable levels with a focus upon new cases. Future development of similar interventions should consider the key areas in the early stages of adoption, before moving towards surveillance and normalisation as part of a wider strategy to achieve long term sustainability and impact [45]. Additionally, this might facilitate further development of the SMASH dashboard with new indicators being successfully introduced over time if these new indicators were acted upon in a similar way and with similar levels of engagement to manage any additional workload.

A key finding of our study illuminates the ways that workflow and work practices were implicated in the integration of the intervention into practice. Since the intervention was pharmacist-led the main users of the dashboard were practice-based pharmacists, but their value was important to the intervention. Pharmacist activity with the dashboard involved responding to the safety signals and verifying the information provided by the SMASH dashboard by reviewing patients’ health records. Beyond these early phases, the decision-making process to resolve cases often involved a collaborative approach with the pharmacist communicating with GPs. In the future similar interventions might utilise pharmacist involvement whilst also providing the support and engagement from practices, particularly in the long term. In light of this it would be of interest to explore how similar interventions might operate as the clinical role of pharmacists evolves, for example in the UK with growing numbers of pharmacists becoming independent prescribers. In this study, pharmacists highlighted barriers to dashboard use that could involve competing work priorities. As the role of clinical pharmacists in general practice evolves, it will be important to explore how their work is integrated with such medication safety dashboards. With the national roll out of prescribing safety indicators as a quality measure the findings here may inform the optimal use in primary care of other dashboards for medication safety and help CCGs and practices to achieve improvements in medication safety [46].

## Conclusions

The utilisation of a mixed-methods approach triangulating qualitative and quantitative data allowed for an in-depth understanding of how the SMASH dashboard was used and integrated into general practice. Our study has highlighted how interactions with the dashboard contributed to making SMASH a sustainable intervention with users, focusing on the most prevalent safety hazards and highest risk (patients highlighted by more than one indicator) first before shifting towards resolving new cases and reducing associated workload In some practices GP staff started to use the dashboard as resource-intensive pharmacist activity reduced. These are important findings since they indicate that the sustainability of such medication safety dashboards may be achieved by processes involving flexible integration into practice where workloads diminish over time, the skills of pharmacists are utilised in early stages and collaborative decision making occurs as the intervention becomes integrated and embedded.

## Supplementary information

**Additional file 1.** Participants involved in the qualitative interviews.

## Data Availability

This study was confined to a single locality (Salford UK). Our ethics approval was granted based on the anonymity of the individuals consenting to participate. For the qualitative data collection participants were drawn from a small group of health care professionals in specific roles, particularly the pharmacists delivering the intervention. Providing additional information beyond the carefully selected anonymised quotations that support the findings in the manuscript, such as additional excerpts from anonymised transcripts, full transcripts or the full data set, would enable others to identify the participants by the way they describe their specific professional roles and associated activities. Furthermore, our ethics approvals were based upon statements in the participant information sheets and consent forms that specifically referred to anonymised quotations from transcripts being used. As such the participants did not consent to information beyond those quotations being made publically available.

## Abbreviations

CDS: Computerised Decision Support
CCG: Clinical Commissioning Group
IT: Information Technology
eGFR: estimated Glomerular Filtration Rate
GP: General Practitioner
SMASH: The Salford Medication safety dASHboard
NOAC: Novel anticoagulants such as apixaban
NSAID: Non-steroidal anti-inflammatory drug
PINCER: The pharmacist-led information technology intervention for medication errors
PPI: Proton-pump inhibitor
SIR: Salford IQR-Integrated Record interquartile ranges
