# Supplementary material for: Understanding the utilisation of a novel interactive electronic medication safety dashboard in general practice: a mixed methods study
Source: BMC Med Inform Decis Mak. 2020 Apr 17;20:69. doi: 10.1186/s12911-020-1084-5 (PMC7164282; doi:10.1186/s12911-020-1084-5)
Supplement: Supplementary file 1 — Additional file 1. Participants involved in the qualitative interviews. [file 12911_2020_1084_MOESM1_ESM.docx]

**Appendix 1.** Participants involved in the qualitative interviews

| **Participant** | **Interviews** | **Role** | **Role within Intervention** |
| --- | --- | --- | --- |
| CCG  Manager1 | 1 | CCG Quality and Improvement Manager | Aligning intervention with other quality and improvement initiatives |
| CCG  Manager2 | 1 | CCG Quality and Improvement Manager | Aligning intervention with other quality and improvement initiatives |
| CCG Pharm | 1 | CCG based pharmacist | Overview of medicines optimisation activities across the Clinical commissioning group  Implementing intervention in three different practices |
| GP1 | 2 | GP | Prescribing lead for practice |
| GP2 | 1 | GP | Prescribing lead for practice |
| GPAdmin1 | 1 | GP Admin- Booking clerk | Administered recall system for patients requiring monitoring |
| PM (Joint interview with PN) | 1 Joint | GP- Practice manager | Overview of medicines safety and quality and improvement initiatives for the practice |
| PN (Joint interview with PM) | 1 Joint | GP - Practice Nurse | Involved in quality and improvement initiatives for the practice |
| GP3 | 1 | GP | No direct involvement with dashboard - communicated with pharmacist |
| GP4 | 1 | GP | No direct involvement with dashboard - communicated with pharmacist |
| P1 | 2 | Practice based pharmacist | Employed by practice - intervention only part of role |
| P2 | 2 | Practice based pharmacist | Employed by practice - intervention only part of role |
| P3 | 2 | Practice based pharmacist | Employed specifically to implement the intervention |
| P4 | 1 | Practice based pharmacist | Employed by practice -intervention only part of role |
| P5 | 1 | Practice based pharmacist | Employed specifically to implement the intervention |
| P6 | 1 | Practice based pharmacist | Neighbourhood Integrated Practice Pharmacist |
| P7 | 1 | Practice based pharmacist with a prescribing qualification | Employed by practice - intervention only part of role |
| P8 | 1 | Practice based pharmacist | Employed by practice - intervention only part of role |
| P9 | 1 | Practice based pharmacist | Neighbourhood Integrated Practice Pharmacist |
| P10 | 1 | Practice based pharmacist | Neighbourhood Integrated Practice Pharmacist |
| P11 | 1 | Practice based pharmacist | Neighbourhood Integrated Practice Pharmacist |
| P12 | 1 | Practice based pharmacist | Employed by practice - intervention only part of role |
